# Hydrolyzed egg yolk peptide prevented osteoporosis by regulating Wnt/β-catenin signaling pathway in ovariectomized rats

**DOI:** 10.1038/s41598-024-60514-8

**Published:** 2024-05-03

**Authors:** Chuanjing Chen, Ludi Huang, Yuanyuan Chen, Jin Jin, Ze Xu, Fei Liu, Kelei Li, Yongye Sun

**Affiliations:** 1 School of Public Health, Qingdao University, Qingdao, China; 2 Institute of Nutrition and Health, School of Public Health, Qingdao University, Qingdao, China; 3 Fine Biotechnological R&D Center, Guangzhou, China

**Keywords:** Hydrolyzed egg yolk peptide (YPEP), Osteoporosis, Ovariectomized (OVX) rats, Wnt/β-catenin, Gut microbiota, Osteoporosis, Nutrition

## Abstract

Hydrolyzed egg yolk peptide (YPEP) was shown to increase bone mineral density in ovariectomized rats. However, the underlying mechanism of YPEP on osteoporosis has not been explored. Recent studies have shown that Wnt/β-catenin signaling pathway and gut microbiota may be involved in the regulation of bone metabolism and the progression of osteoporosis. The present study aimed to explore the preventive effect of the YPEP supplementation on osteoporosis in ovariectomized (OVX) rats and to verify whether YPEP can improve osteoporosis by regulating Wnt/β-catenin signaling pathway and gut microbiota. The experiment included five groups: sham surgery group (SHAM), ovariectomy group (OVX), 17-β estradiol group (E2: 25 µg /kg/d 17β-estradiol), OVX with low-dose YPEP group (LYPEP: 10 mg /kg/d YPEP) and OVX with high-dose YPEP group (HYPEP: 40 mg /kg/d YPEP). In this study, all the bone samples used were femurs. Micro-CT analysis revealed improvements in both bone mineral density (BMD) and microstructure by YPEP treatment. The three-point mechanical bending test indicated an enhancement in the biomechanical properties of the YPEP groups. The serum levels of bone alkaline phosphatase (BALP), bone gla protein (BGP), calcium (Ca), and phosphorus (P) were markedly higher in the YPEP groups than in the OVX group. The LYPEP group had markedly lower levels of alkaline phosphatase (ALP), tartrate-resistant acid phosphatase (TRAP) and C-terminal telopeptide of type I collagen (CTX-I) than the OVX group. The YPEP groups had significantly higher protein levels of the Wnt3a, β-catenin, LRP5, RUNX2 and OPG of the Wnt/β-catenin signaling pathway compared with the OVX group. Compared to the OVX group, the ratio of OPG/RANKL was markedly higher in the LYPEP group. At the genus level, there was a significantly increase in relative abundance of *Lachnospiraceae_NK4A136_group* and a decrease in *Escherichia_Shigella* in YPEP groups, compared with the OVX group. However, in the correlation analysis, there was no correlation between these two bacteria and bone metabolism and microstructure indexes. These findings demonstrate that YPEP has the potential to improve osteoporosis, and the mechanism may be associated with its modulating effect on Wnt/β-catenin signaling pathway.

## Introduction

Osteoporosis (OP) is a systemic bone disease characterized by reduced bone mass and destruction of the microstructure of the bone tissue, which could increase the risk of fracture^1^. More than 200 million individuals are impacted by osteoporosis worldwide^2^. The number of osteoporosis patients in China is expected to reach 120 million by 2050^3^. Conventional therapeutic agents for osteoporosis include bisphosphonate, estrogen replacement therapy, calcitonin and fluoride^4^. However, these agents failed to restore bone turnover equilibrium or had multiple adverse effects after long-term usage^5^. Therefore, there is an urgent need to discover new effective natural substances to play an anti-osteoporosis role with less side effects.

There is growing evidence that bioactive peptides can promote bone health^6–9^. Hydrolyzed egg yolk peptide (YPEP) is a bioactive peptide extracted from egg yolk. Up to now, only a few studies researched the beneficial role of YPEP on prevention or treatment of osteoporosis. An in vitro experiment found that YPEP promoted osteoblast proliferation and differentiation by activating the MAPK/ ERK1 signaling pathway^10^. In addition, YPEP was shown to increase bone mineral density (BMD) in ovariectomized (OVX) rats^11^. However, the underlying mechanism of YPEP on osteoporosis has not been explored in vivo.

Wnt/β-catenin signaling plays an important role in the regulation of bone mass and strength^12^. Activation of this canonical pathway depends on the binding of Wnt, frizzled receptors and low-density lipoprotein receptor-related protein 5/6 (LRP5/6) co-receptor. The binding inhibits the expression of glycogen synthase kinase-3β (GSK3β) and stabilizes the accumulation of β-catenin in nucleus of cells, thereby promoting its nucleus translocation and stimulating the proliferation and differentiation of osteoblasts^13,14^. Previous studies^15,16^ have demonstrated that various bioactive peptides can facilitate osteogenesis by activating the Wnt/β-catenin signaling pathway to improve osteoporosis. Based on the osteogenic efficacy of YPEP observed in previous investigation, we postulate that YPEP can ameliorate osteoporosis in OVX-induced rats through activating the Wnt/β-catenin signaling pathway.

The gut-bone axis has gained much focus of researches as a target for exploring the relationship between food composition and bone metabolism^17^. The metabolites of intestinal flora such as lipopolysaccharide (LPS), can induce intestinal inflammatory reactions, then lead to changes in bone mass and bone structure^18^. Elevated concentrations of *Lactobacillus reuteri* and *Bifidobacterium longum* in the gut may increase BMD by promoting the absorption calcium^19^. For the past few years, it has been found that the gut microbiota may exert significant effects on 5-hydroxytryptamine (5-HT) signaling^20^. The 5-HT signal transduction system is considered a vital factor in regulating bone development and health^21^. An increasing body of evidence suggests that bioactive peptides can regulate gut microbiota in various chronic metabolic diseases^22,23^. As a bioactive peptide produced during fermentation of milk, kefir peptide can modulate the structure of the gut microbiota to prevent menopausal osteoporosis in OVX mice^24^. Although no relevant studies have been conducted, we hypothesize that YPEP may play a role in ameliorating osteoporosis by regulating the composition of gut microbiota.

The purpose of this study was to research the effects of YPEP on bone protection in OVX-induced rats. The effects of YPEP on the Wnt/β-catenin signaling pathway and gut microbiota in OVX rats were evaluated to interpret the underlying mechanism.

## Material and method

### Materials and reagents

YPEP is provided by Hunan Fine Biotechnology Co., Ltd. (Changsha, China). ALP(JM-01624R1), BALP (JM-02025R1), BGP (JM-02028R1), TRAP (JM-02026R1) and CTX-I (JM-02171R1) ELISA kits were received from Jingmei Biotechnology Co., Ltd. (Yancheng, Jiangsu, China). RIPA lysis buffer (R0010) and PMSF (P0100) were received from Soleibao Biotechnology Co., Ltd. (Beijing, China). Phosphatase inhibitor (MB2678) and enhanced chemiluminescence (ECL, MA0186) were obtained from Meilun Biotechnology Co., Ltd. (Dalian, China). The BCA protein quantitative kit (MA0082) was obtained from Yase Biotechnology Co., Ltd. (Shanghai, China). The primary antibody of Wnt3a (ab219412), β-catenin (ab32572), LRP5 (ab223203), RUNX2 (ab236639) of Rabbit-anti-rat and the secondary antibody of goat anti-rabbit IgG antibody-HRP (ab288151) were obtained from Abcam Biosciences Co., Ltd. (New York, New Jersey, USA). β-actin (GB11001-100) was purchased from Seville Biotechnology Co., Ltd. (Wuhan, Hubei, China). The primary antibody of OPG (abs155185) and RANKL (abs155193) were purchased from Absin Biosciences Co., Ltd. (Shang Hai, China).

### Molecular weight (MW) distribution of YPEP

The molecular weight distribution of YPEP was determined using a Shimadzu LC-20A high-performance liquid chromatography (HPLC) system (Shimadzu, Tokyo, Japan), equipped with a Shodex Asahi pak GS-320 HQ column (Tosoh, Tokyo, Japan). The samples were loaded onto the HPLC and eluted with 40% (v/v) acetonitrile containing 5% (v/v) trifluoroacetic acid at a flow rate of 0.5 mL/min. The detection was performed at room temperature using an ultraviolet detector set at 214 nm.

### Amino acid composition of YPEP

According to the national standard of the People’s Republic of China GB5009.124-2016, 10 mg YPEP was accurately weighed and added to 10 ml solution of hydrochloric acid (6 mol/L). Subsequently, 1 ml phenol solution were introduced into the hydrolysis tube. Then the hydrolyzed tube was frozen in a refrigerant (salt mixed with ice cubes at a mass of 1:3) for 4 min, followed by vacuuming and nitrogen blowing procedures (repeated three times before sealing). Then the sealed tube was put in a furnace at a temperature of 110 °C for 22 h, after which it was removed from the furnace and allowed to cool down until reaching room temperature. The hydrolysate was filtered into a 50 ml volumetric bottle, and the hydrolysate container was rinsed multiple times with a small amount of water. The resulting rinse solution was then transferred into the same volumetric bottle, ensuring a final volume of 50 ml. The filtrate (1.0 ml) was transferred to a 15 ml tube and dried with a tube concentrator at a reduced pressure of 40 °C. Dried residue was dissolved in water (2 ml) and dried again under reduced pressure. Subsequently, dissolve the sample with sodium citrate at pH 2.2. After the solution is thoroughly mixed, it is filtered with a 2.2 µm filter membrane and then transferred to the sample bottle of the instrument. The mixed amino acid standard working medium and sample determination liquid were injected into the HITACHI L-8900 automatic amino acid analyzer (Tokyo, Japan) in equal volumes respectively, and analyzed it in accordance with the JJG1046-2011 amino acid analyzer verification regulations and instrument instructions.

### Determination of amino acid sequence

First, the YPEP sample was dissolved in 50 mmol/L NH_4_HCO_3_, after reduced by 10 mmol/L dithiothreitol at 56 °C for 1 h, then alkylated by 55 mmol/L iodoacetamide at room temperature in dark for 40 min. The self-filling C18 column was used for desalting and the solvent was dried in a vacuum centrifugal concentrator at 45 °C. Then, the dried product was dissolved with 20 µL 0.1% formic acid and analyzed by LC–MS/MS. The conditions for liquid chromatography were as follows: pre-column, 150 µm i.d. ×5 cm, packing:Reprosil-Pur 120 C18-AQ 3 µm; analytical column, 100 µm i.d. ×180 mm, packing: Reprosil-Pur 120 C18-AQ 3 µm; mobile phase A, 0.1% formic acid; mobile phase B, a mixture of formic acid and acetonitrile (1/4, w/w); total flow rate, 600 nL/min; The LC linear gradient lasted from 4 to 8% mobile phase B for 2 min, from 8 to 28% mobile phase B for 43 min, from 28 to 40% mobile phase B for 10 min, from 40 to 95% mobile phase B for 1 min, and from 95 to 95% mobile phase B for 10 min. Parameters of MS conditions were as follows: spray voltage, 2.2 kV; capillary temperature: 270 °C; MS resolution, 70,000 at 400 m/z; MS precursor m/z range, 300–1800 m/z. The raw MS files were analyzed and searched against protein database based on the species of the samples using Byonic.

### Animal experiment design

Female eight-week-old Sprague–Dawley rats were obtained from Sipford Biotechnology Co., LTD (Beijing, China). Each subject was individually housed under SPF conditions at a temperature of 23 ± 1 °C with a 12-h light/dark cycle. They had a libitum access to food and water throughout the feeding experiment. After a 7-day adaptation period, the rats underwent bilateral ovariectomy surgery (OVX, n = 40), or bilateral laparotomy to maintain intact ovaries as a sham control group (SHAM, n = 10). The OVX rat model is well established in investigations of osteoporosis and osteoporotic therapies^25^. Ovariectomy cuts off the secretion of estrogen, resulting in a sharp decrease in estrogen production in rats, so we chose estrogen as a positive intervention. All rats received daily penicillin injections for three days and had a 14-day postoperative recovery period. On day 15 after ovariectomy, the OVX rats were randomly assigned into 4 groups (n = 10): bilateral ovariectomy group (OVX), estrogen intervention group (E2), low dose YPEP group (LYPEP) and high dose YPEP group (HYPEP), and they were given normal saline, 25 µg/kg/d 17β-estradiol, 10 mg/kg/d YPEP and 40 mg/kg/d YPEP by gavage, respectively. Body weight was assessed on a weekly basis. Following 8 weeks of treatment, rats were anesthetized (1% pentobarbital sodium, 0.4 ml/100 g, i.p.) and sacrificed after a 12-h fast to obtain blood and tissues samples. The blood was centrifuged at 3500 rpm and 4 °C for 10 min to isolate serum, which was subsequently stored at − 80 °C for further analysis. The left femur and tibia were fixed in 4% paraformaldehyde for histomorphology and BMD micro-CT analysis. The remaining bone was preserved at − 80 °C for other analysis. Cecal contents were collected from each rat aseptically. The present study was granted approval by the Laboratory Animal Welfare Ethics Committee of Qingdao University (QDU-ACE-2022489). According to local legislation and system requirements, we implement animal testing procedures in accordance with the experimental animal care guidelines of Qingdao University, and all procedures comply with the standards outlined in the ARRIVE guidelines.

### Determination of bone turnover indicators in serum

The serum levels of bone turnover markers, including bone gla protein (BGP), alkaline phosphatase (ALP), bone alkaline phosphatase (BALP), tartrate-resistant acid phosphatase (TRAP) and C-terminal telopeptide of type I collagen (CTX-I) were quantified using rat ELISA kits. The calcium (Ca) and phosphorus (P) concentrations in serum were measured by an automatic biochemical analyzer.

### Micro-CT scanning

The distal femur was vertically scanned using a high-resolution microscopic CT scanner (Quantum GX2, PerkinElmer, Japan). The camera resolution was 10 µm, the source voltage was 50 kV, and the source current was 100 µA. The single scan time was 2 min. The region of interest (ROI) for each femur was defined as a 1 mm boundary near the proximal growth plate. The trabecular network was directly performed in the dimensions using supporting analysis software to calculate quantitative data, including tissue volume (TV), bone volume (BV), bone volume fraction (BV/TV), trabecular number (Tb.N), trabecular thickness (Tb.Th) and trabecular separation (Tb.Sp).

### Femoral ash

The empty porcelain boat was placed in a muffle furnace (SX2-4-10TP, Shanghai Yiheng Scientific Instrument, China) and heated to 600 °C. After cooling, the weight of the boats was measured and recorded. Subsequently, the rat femurs were placed in the porcelain boat, heated at 105 °C for 4–6 h, and then placed in a dryer to cool for 30 min. The boat was then weighed. The sample was placed in a muffle oven with the temperature set to 200 °C. The temperature was incrementally raised by intervals of 100 °C until reaching a maximum of 700 °C during heating gradient stages. Finally, after cooling down, the sample underwent another weighing process.

### Three-point bending test

The mechanical properties of the femurs were measured by three-point test using electronic universal material testing machine (WXR130 Jiangsu, China). The upper loading device was aligned to the center of the femoral shaft. The distance between the points supporting the femurs was 18 mm, and the displacement rate was 0.03 mms^−1^.

### Histopathological changes

The bone tissues were fixed in neutral-buffered 10% formaldehyde solution for 24-h. Subsequently, the femur was decalcified with a 10% tetraacetic acid (EDTA) solution (pH 7.4) at 4 °C for 4 weeks. After gradient alcohol dehydration, the specimens were embedded in paraffin wax and sectioned continuously into 4 µm thick slices, which were then stained with hematoxylin and eosin (H&E) and TRAP. The sections were observed under an optical microscope (Olympus, Tokyo, Japan). TRAP staining revealed that the cytoplasm of osteoclasts appeared wine red while their nuclei displayed a light blue. The region of interest observed in each femur was defined as a 1 cm boundary near the proximal growth plate. In the process of image collection, the researcher was blinded.

### Western blot analysis

The femoral metaphysis (100 mg) was pulverized in liquid nitrogen, followed by homogenization in cold RIPA lysis buffer and centrifugation to collect the supernatant. The proteins were quantified using a BCA protein assay kit. Subsequently, 40 µg protein was loaded onto 8 or 10% SDS–PAGE and then moved to a polyvinylidene difluoride (PVDF) membranes. Then, the membranes were blocked and incubated overnight at 4 °C with the appropriate primary antibody [Wnt3a (1:1000), β-catenin (1:1000), LRP5 (1:1000), RUNX2 (1:1000), OPG (1:1000) and RANKL (1:1000)]. The primary antibody reaction membranes were washed with TBST and subsequently incubated with the corresponding HRP-labeled secondary antibody at room temperature for 90 min. The protein bands were observed by autoradiography using an enhanced chemiluminescence (ECL) localization reagent, and subsequently detected and quantified with Image J software (National Institutes of Health, USA).

### Sequencing of gut microbiota

Rat fecal DNA was extracted strictly on the basis of the operational steps of a QIAamp Fast DNA stool Mini Kit (Qiagen, Hilden, Germany). The target fragments were then recovered using a gel extraction kit. The purified PCR products were quantified and homogenized using a real-time fluorescence quantitative PCR instrument to form a sequencing library. The quality of the library was inspected by agarose gel electrophoresis and microplate reader, with Qubit used for quantitative analysis after passing the test. Illumina Novaseq 6000 platform was employed for high-throughput sequencing.

### Statistical analysis

The statistical software packages SPSS 26.0, GraphPad Prism 8.0 and Figdraw^2^ were utilized for data analysis and visualization. The data were presented as mean ± SD or mean ± SEM. One-way analysis of variance (ANOVA) and LSD post-hoc tests were used to analyze differences between groups. Alpha diversity of gut microbiota was assessed by the Chao1 index, ACE index, Shannon index, and Simpson index. Beta diversity of gut microbiota was evaluated by principal coordinates analysis (PCoA) based on binary Jaccard. In the univariate analysis of gut microbiota in each group, one-way analysis of variance (ANOVA) was used, and *p* values were adjusted for multiple comparison using the Benjamini–Hochberg false discovery rate (FDR). The correlations between gut microbiota and parameters of bone metabolism were assessed by Spearman analysis. *P* < 0.05 was considered statistically significant.

## Results

### Characterization of YPEP

The distribution of molecular weight for hydrolyzed egg yolk peptide was primarily concentrated below 1000 Da. (see Supplementary Fig. S1 online).

A total of 17 distinct amino acids were identified in the hydrolyzed peptides derived from egg yolk (Table 1). Glutamic acid (Glu) emerged as the predominant amino acid within YPEP, while Aspartic acid (Asp), Serine (Ser), Alanine (Ala), Leucine (Leu), Arginine (Arg), and Lysine (Lys) were also found to be abundant constituents of YPEP. Conversely, Histidine (His), Methionine (Met), Glycine (Gly), and Cysteine (Cys) exhibited the lowest levels among all detected amino acids within YPEP.

**Table 1:** Amino acid compositions of hydrolyzed egg yolk peptide.

Amino acid	Relative content ^1^ (g/100 g)	Amino acid	Relative content (g/100 g)
Asp	9.49 ± 0.18	Met	2.35 ± 0.35
Thr	4.83 ± 0.09	Ile	4.58 ± 0.15
Ser	6.50 ± 0.14	Leu	7.60 ± 0.13
Glu	12.51 ± 0.24	Tyr	4.64 ± 1.38
Gly	3.12 ± 0.06	Phe	3.90 ± 0.70
Ala	5.10 ± 0.08	Lys	6.34 ± 0.12
Cys	1.43 ± 0.23	His	2.05 ± 0.48
Val	5.52 ± 0.09	Arg	6.24 ± 0.12
Pro	4.28 ± 0.16		

^1^Relative content of individual amino acids was expressed as g per 100 g of the total amino acids in hydrolyzed egg yolk peptide. Values were shown as the mean ± SD.

The raw files collected by MS were searched through the Byonic database to obtain the identification results. Table 2 showed the top 10 peptides with high scores. The peptide sequences are as follows: L.AHSPIIK.V, S.SAADIPVHIQ.I, A.PGHGIDK.L, M.TPPLTGDF.R, E.FDEKPADLPSLVEK.Y, E.GSGGTAVTGR.V, Q.VWYGPDEKIPSIR.R, R.MSFKPVYSDVPIEK.I, E.TQPGVLRPGLQ.S, A.DTDSVRPR.V.

**Table 2:** The top 10 peptides with high scores in hydrolyzed egg yolk peptide.

Peptide	Observed m/z	z	Observed (M + H)	Calc.mass (M + H)	Mass error (ppm)	Score	Scan time	Intensity
L.AHSPIIK.V	383.236	2	765.465	765.462	3.7	524.8	10.0280	26,833,000
S.SAADIPVHIQ.I	525.787	2	1050.566	1050.558	7.7	490.3	28.0435	436,900,000
A.PGHGIDK.L	362.193	2	723.378	723.378	− 0.4	486.2	6.6581	38,023,000
M.TPPLTGDF.R	847.420	1	847.420	847.420	0.2	477.5	35.8794	39,392,000
E.FDEKPADLPSLVEK.Y	794.421	2	1587.834	1587.827	4.7	477.3	33.3137	346,210,000
E.GSGGTAVTGR.V	431.725	2	862.442	862.438	4.5	467.1	6.8512	9,653,300
Q.VWYGPDEKIPSIR.R	520.616	3	1559.834	1559.822	7.7	439.4	33.9310	22,260,000
R.MSFKPVYSDVPIEK.I	547.291	3	1639.858	1639.840	10.9	439.2	33.2936	131,280,000
E.TQPGVLRPGLQ.S	583.339	2	1165.671	1165.669	1.4	438.3	24.6102	62,311,000
A.DTDSVRPR.V	473.242	2	945.476	945.475	1.4	437.2	7.5975	24,204,000

Glycine: G; Alanine: A; Valine: V; Leucine: L; Isoleucine: I; Proline: P; Phenylalanine: F; Tyrosine: Y; Tryptophan: W; Serine: S; Threonine: T; Methionine: M; Glutarnine: Q; Asparticacid: D; Glutamicacid: E; Lysine: K; Arginine: R; Histidine: H.

### Weight and food intake of rats

**Figure 1:**
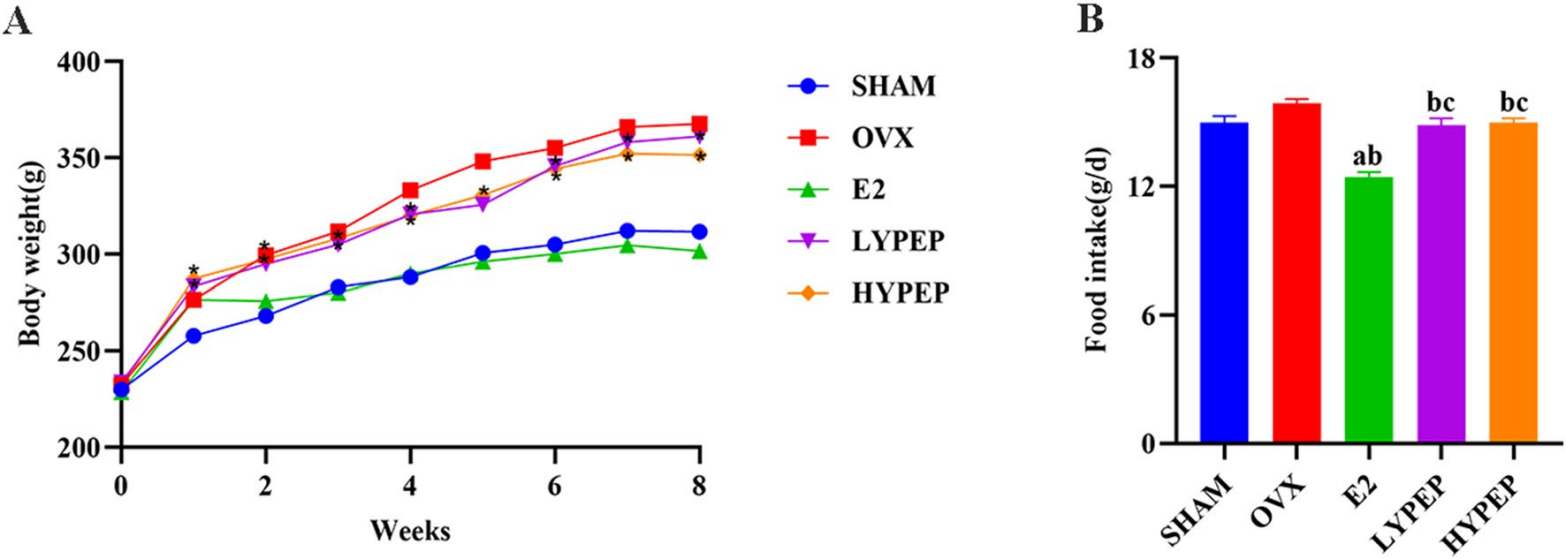
Effects of YPEP on the weight and food intake. (**A**) Wight changes during the 8-week experiment; (**B**) food intake. Results are presented as means ± standard error of the mean (SEM), (n = 8). * *p* < 0.05, compared to the OVX group. a *p* < 0.05, compared to the SHAM group; b *p* < 0.05, compared to the OVX group; c *p* < 0.05, compared to the E2 group.

There were no significant differences in the initial weight of rats among each group. The weight of rats in OVX group, LYPEP group and HEPYP group was higher than that in SHAM group (*p* < 0.05) (Fig. 1A). The feed intake of E2 group was significantly lower than that in the other four groups. Additionally, we found that both LYPEP and HEPYP groups had a significantly lower feed intake compared with the OVX group (*p* < 0.05) (Fig. 1B).

### YPEP reversed the damage to bone microstructure

**Figure 2:**
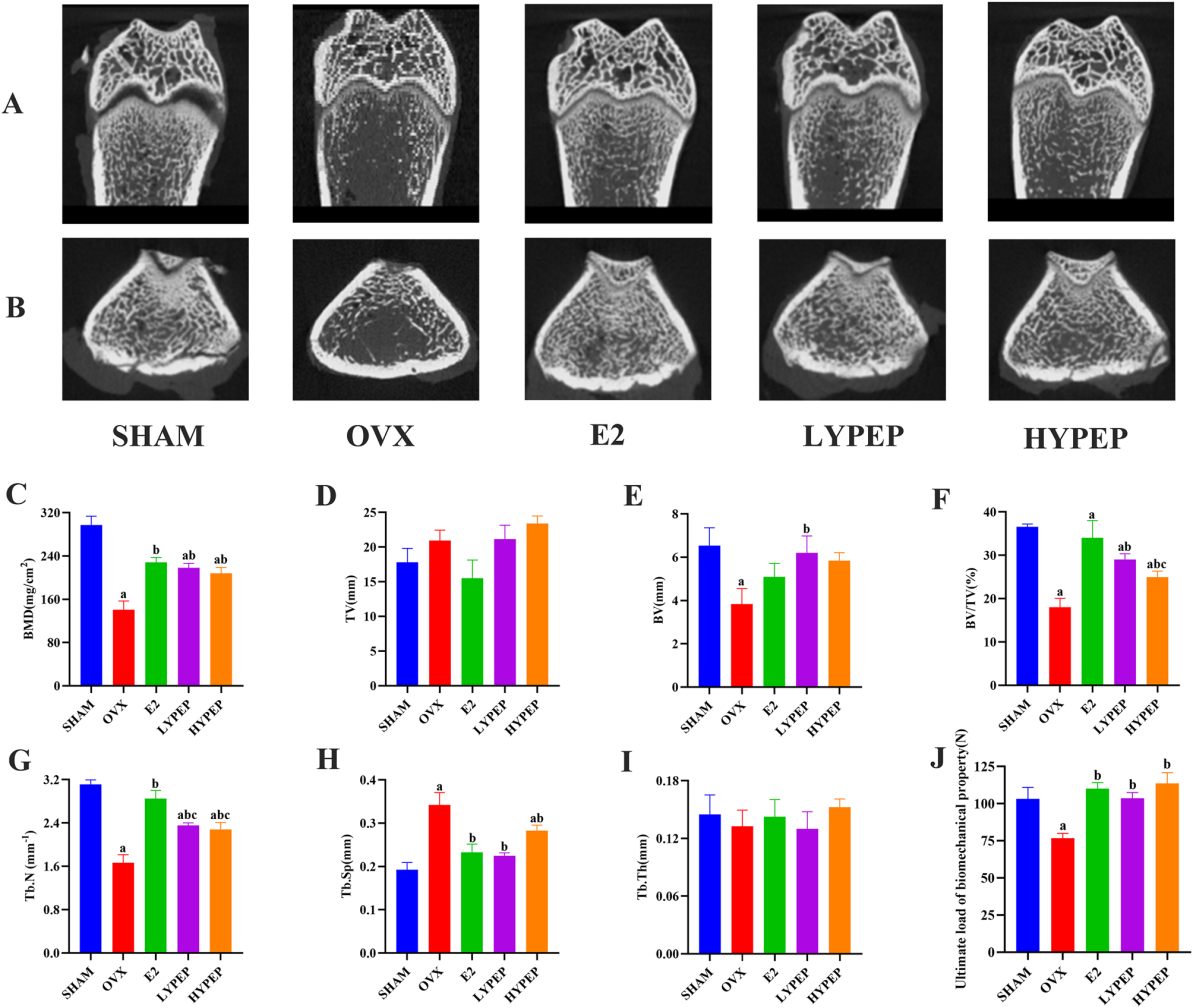
3D reconstruction images, bone microstructure parameters of the metaphysis of distal femur and mechanical properties in rats. (**A**) 3D reconstruction of the metaphyseal of longitudinal section of the distal femur. (**B**) 3D reconstruction of the metaphyseal of cross section of the distal femur. (**C**) Bone mineral density. (**D**) Tissue volume. (**E**) Bone volume. (**F**) Bone volume fraction. (**G**) Trabecular number. (**H**) Trabecular separation. (**I**) Trabecular thickness. (**J**) Ultimate load of biomechanical property. Results are presented as means ± standard error of the mean (SEM), (n = 4). a *p* < 0.05, compared to the SHAM group. b *p* < 0.05, compared to the OVX group; c *p* < 0.05, compared to the E2 group.

After conducting three-dimensional reconstruction of the distal femora, it was observed that bone microarchitecture seriously damaged in the OVX group, compared to the SHAM group (Fig. 2). The BMD, BV, BV/TV, and Tb.N were significantly lower (*p* < 0.05) in OVX group than in the SHAM group. The OVX group had a significantly higher Tb.Sp than the SHAM group. The YPEP groups had significantly higher BV, BV/TV and Tb.N than the OVX group. In contrast, the LYPEP and HYPEP groups had a significantly lower Tb.Sp than in the OVX group (*p* < 0.05).The results of the three-point bending test showed that the mechanical properties of in the OVX group were weakened, and the maximum load of the femurs in the OVX group was significantly lower than in the LYPEP and HYPEP groups (Fig. 2J).

**Figure 3:**
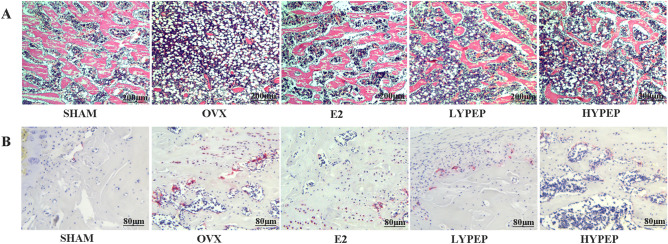
Effects of YPEP on the Hematoxylin–Eosin (H&E) and Tartrate-resistant acid phosphatase (Trap) staining (magnification, ×40 and ×100; scale bars, 200 µm and 80 µm) of histologically sectioned distal femur tissue of OVX rats, (n = 3).

In comparison to the SHAM group, the distal femoral canal of the OVX group was infiltrated with adipocytes (Fig. 3A). Administration of E2, LYPEP and HYPEP effectively ameliorated these unfavorable alterations in trabecular microstructure; adipocyte accumulation decreased significantly. The OVX group had significantly more osteoclast than the SHAM, E2, LYPEP and HYPEP groups (Fig. 3B).

### YPEP supplementation inhibited serum high bone turnover in OVX rats

**Figure 4:**
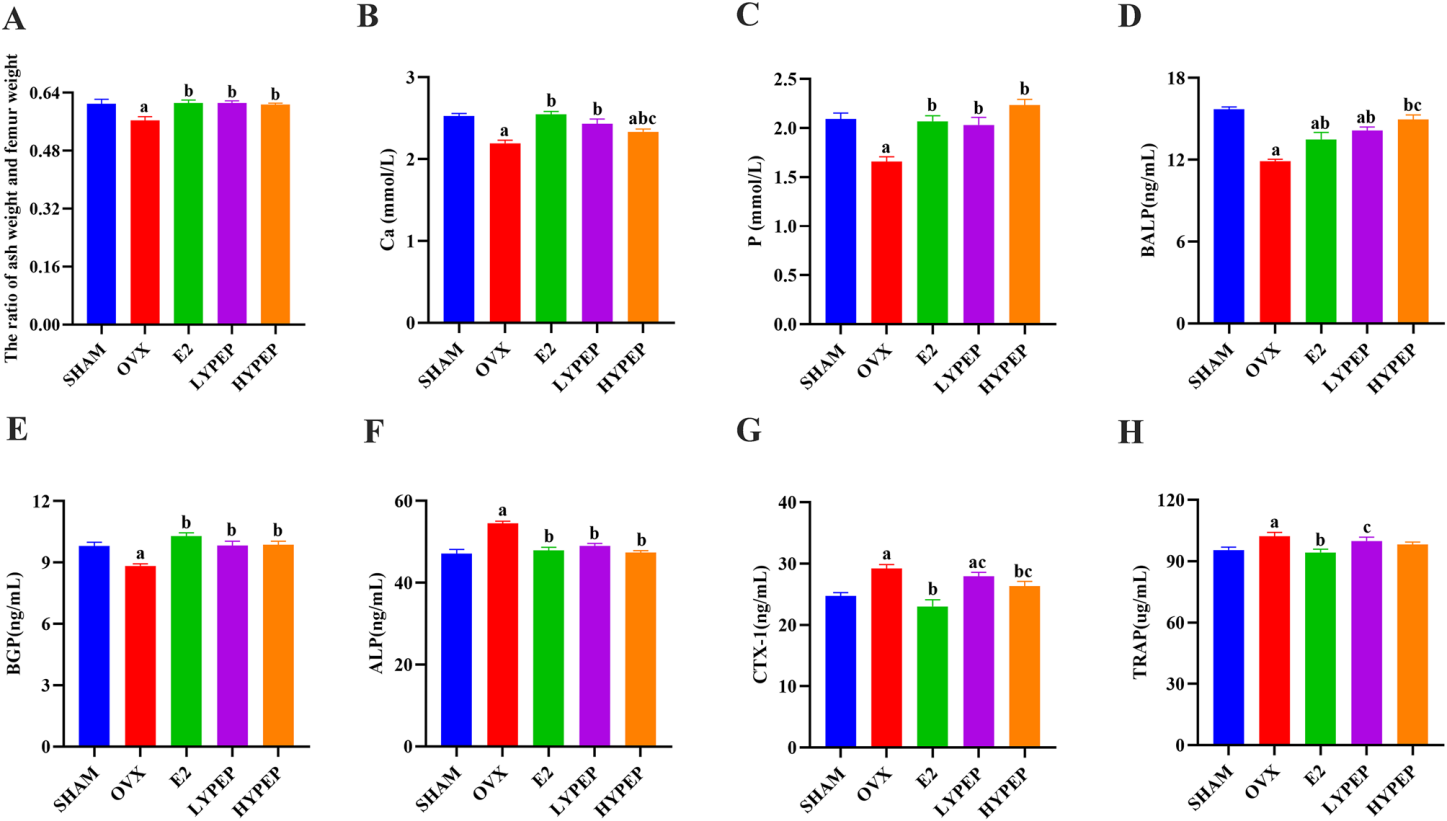
Effects of YPEP on mineral content and serum bone turnover markers. (**A**) The ratio of ash weight and femur weight. (**B**) Serum of Ca concentration. (**C**) Serum of P concentration. (**D**) Serum of BALP concentration. (**E**) Serum of BGP concentration. (**F**) Serum of ALP concentration. (**G**) Serum of CTX-I concentration. (**H**) Serum of TRAP concentration. Results are presented as means ± standard error of the mean (SEM) (n = 4 or 7). a *p* < 0.05, compared to the SHAM group. b *p* < 0.05, compared to the OVX group; c *p* < 0.05, compared to the E2 group.

The relative of the ash and femur weight was significantly lower in the OVX group than in the SHAM group. YPEP groups had a significantly higher relative of the ash and femur weight than the OVX group (Fig. 4A). Serum levels of Ca and P were markedly lower in the OVX group than in the SHAM group (*p* < 0.05). Compared to the OVX group, the E2, LYPEP, and HYPEP groups had markedly higher levels of serum Ca and P (*p* < 0.05) (Fig. 4B,C).

Compared to the SHAM group, the OVX group had significantly lower levels of BALP and BGP in serum. The E2, LYPEP, and HYPEP groups had significantly higher BGP and BALP levels than the OVX group. The OVX group had markedly higher levels of ALP, CTX-I and TRAP than the SHAM group. The serum ALP and CTX-I levels were significantly lower in E2 and YPEP groups than those in the OVX group (Fig. 4).

### YPEP regulated the Wnt/β-catenin signaling in OVX rats

**Figure 5:**
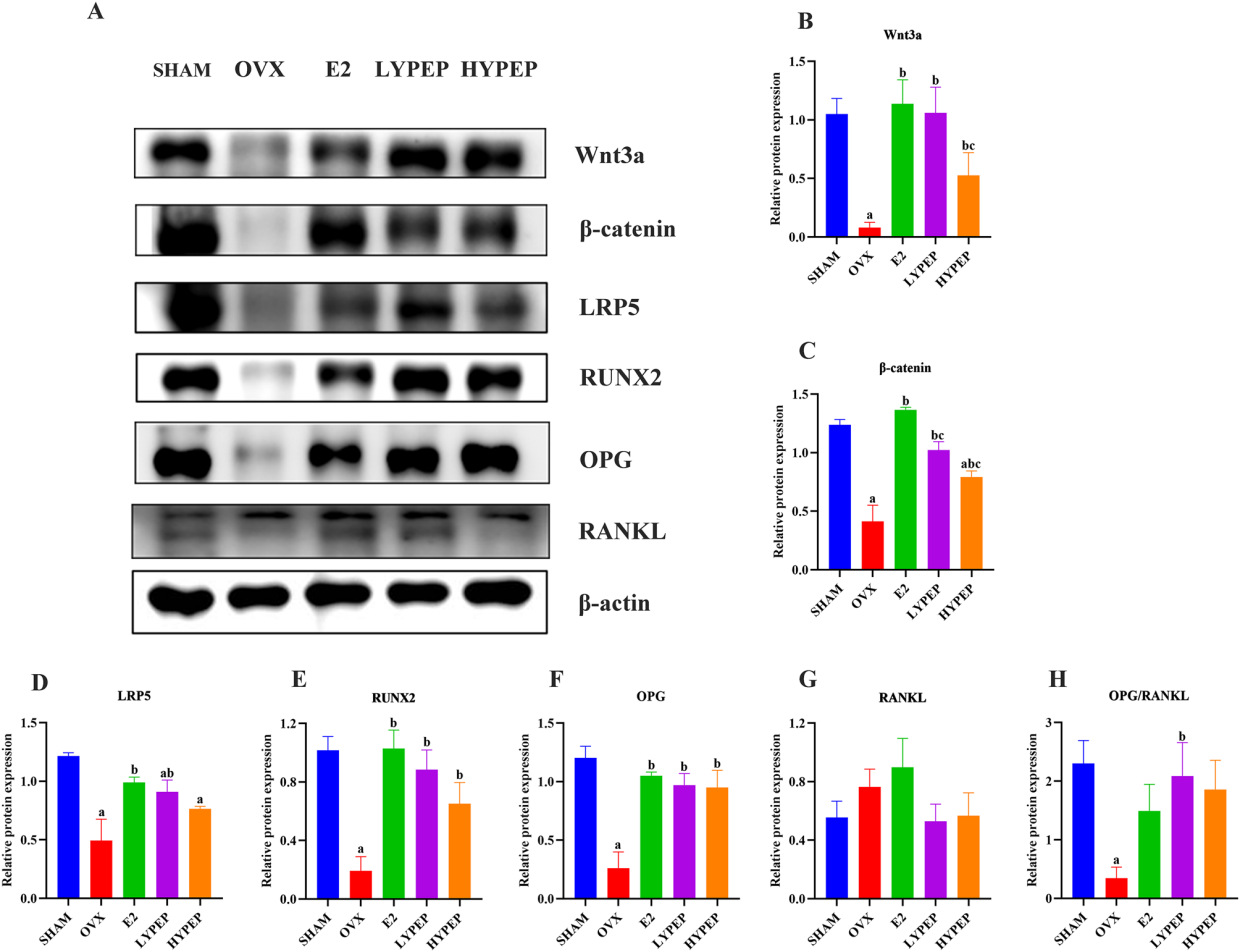
Western blot analysis on the key genes in the Wnt/β-catenin signaling pathway. (**A**) Representative images of western blotting for Wnt3a, β-catenin, LRP5, RUNX2, OPG, RANKL and using β-actin as the loading control. (**B**–**H**) quantitative analyses of the protein of Wnt3a, β-catenin, LRP5, RUNX2, OPG, RANKL and the ratio of OPG to RANKL. Results are presented as means ± standard error of the mean (SEM) (n = 3). The gel images presented in this paper are cropped, and the original gel images are provided in Supplementary Fig. 2. a *p* < 0.05, compared to the SHAM group. b *p* < 0.05, compared to the OVX group; c *p* < 0.05, compared to the E2 group.

The protein levels of Wnt3a, β-catenin, LRP5 and RUNX2 were significantly lower in the OVX group than in the SHAM group. The E2, LYPEP and HYPEP groups had significantly higher protein levels of Wnt3a, β-catenin, LRP5 and RUNX2 compared to the OVX group, while no significant difference of the protein levels of LRP5 between the OVX and the HYPEP groups (Fig. 5) (*p* < 0.05).

The OVX group had a memorably lower protein level of OPG than the SHAM group. Compared to the OVX group, the E2, LYPEP and HYPEP groups had a memorably higher protein level of OPG (*p* < 0.05). There was no significant difference in RANKL levels among the five groups. The ratio of OPG/RANKL was significantly higher in the LYPEP group than in the OVX group, while no significant difference in the ratio of OPG/RANKL between the OVX and the SHAM groups (Fig. 5).

### YPEP supplementation altered gut microbial structure

**Figure 6:**
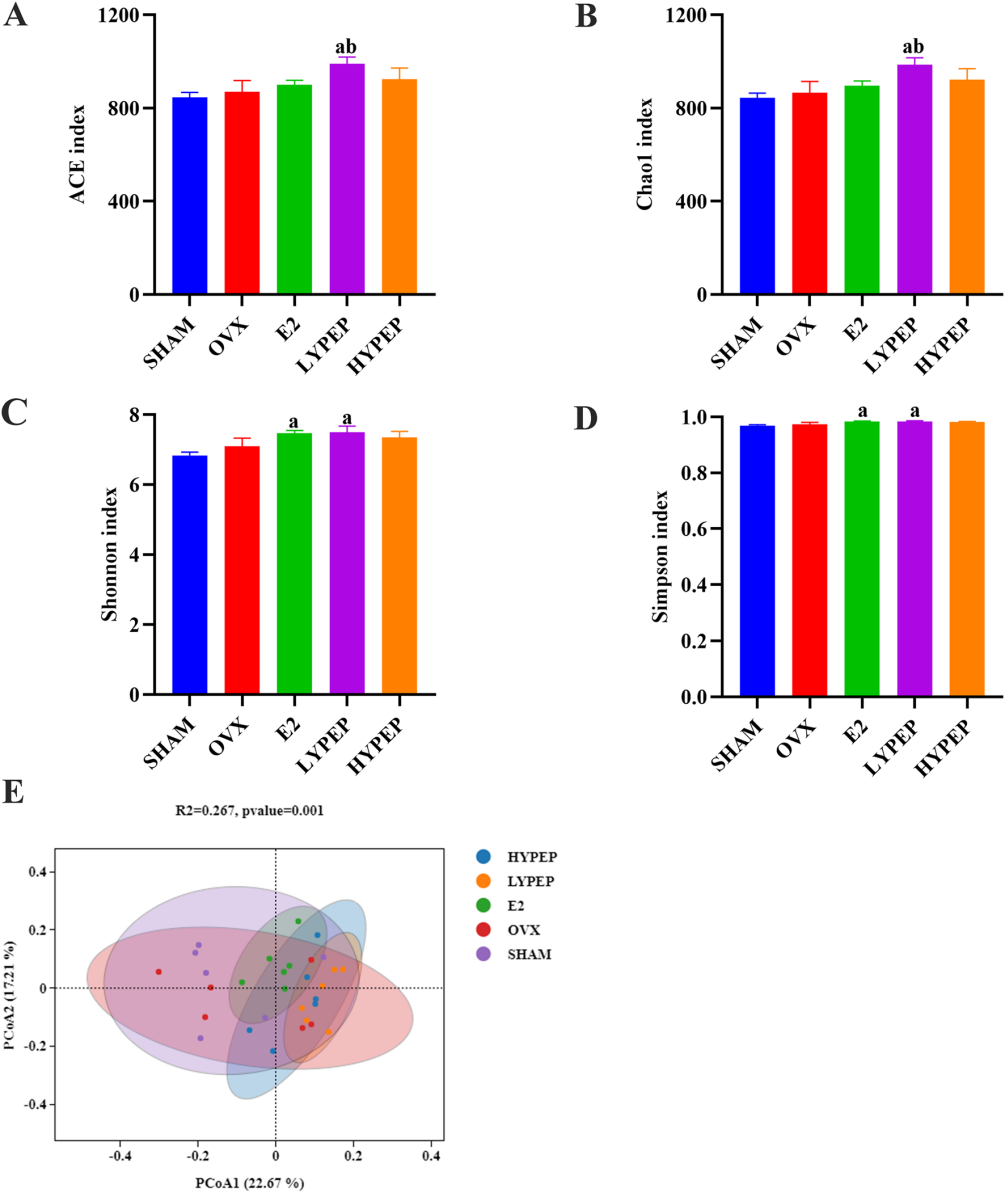
Effect of YPEP on alpha and beta diversity of gut microbiota of rats. (**A**) ACE index; (**B**) Chao 1 index; (**C**) Shannon index; (**D**) Simpson index. (**E**) PCoA at the genus level. Results are presented as means ± standard error of the mean (SEM), (n = 6). a *p* < 0.05, compared to the SHAM group. b *p* < 0.05, compared to the OVX group; c *p* < 0.05, compared to the E2 group.

The Chao1 index and ACE index in the LYPEP group were significantly higher than those in the SHAM and OVX group (Fig. 6A,B). The Shannon and Simpson indexes were significantly higher in E2 and LYPEP groups than SHAM and OVX groups (Fig. 6C,D). Significant group difference was observed in PCoA analysis (Fig. 6E).

**Figure 7:**
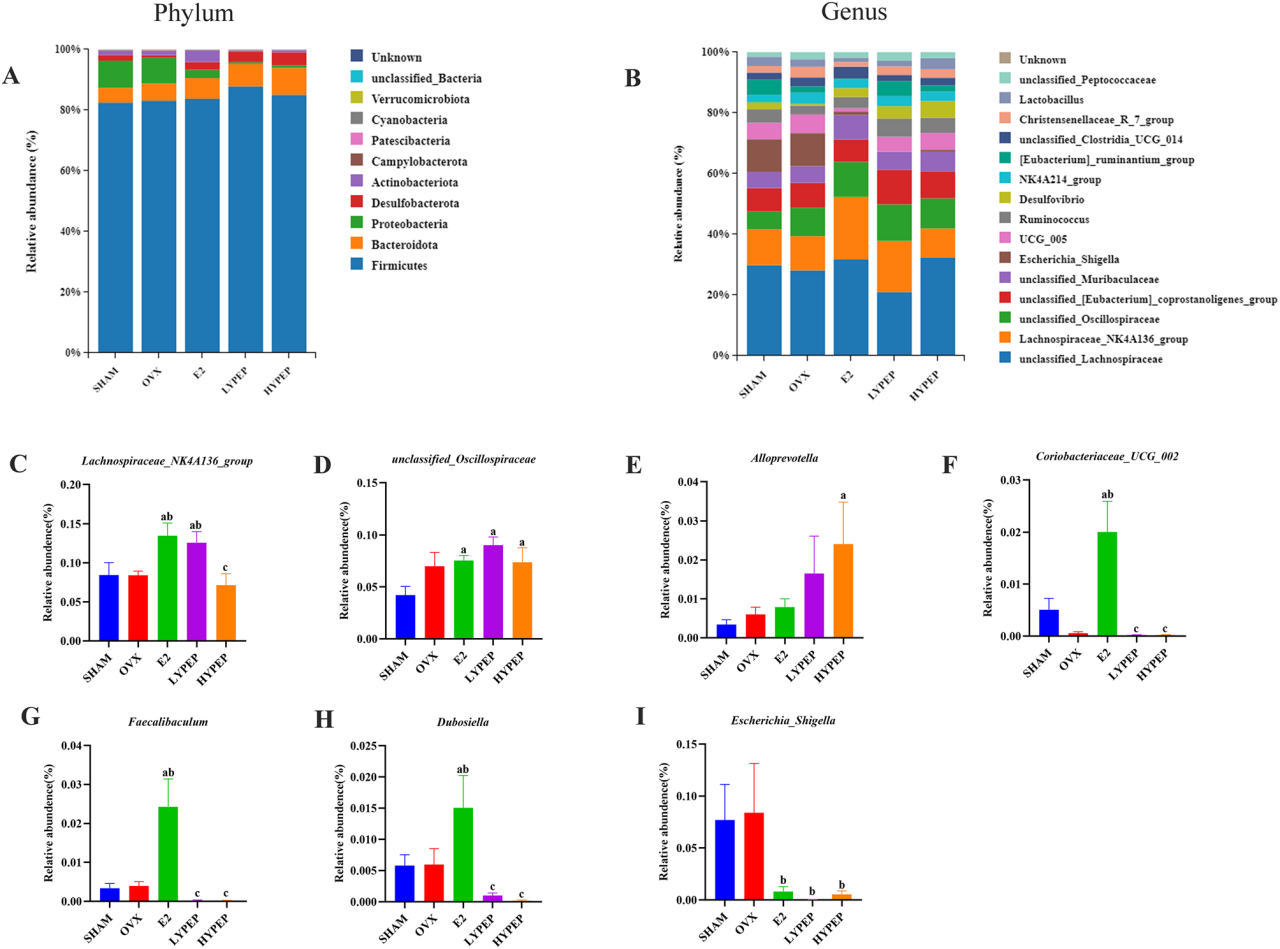
Effect of YPEP on species relative abundance of gut microbiota. (**A**) The microbial distributions at the phylum level. (**B**) The microbial distributions at the genus level. The relative abundance of (**C**) *Lachnospiraceae_NK4A136_group* (**D**) *unclassified_Oscillospiraceae* (**E**) *Alloprevotella* (**F**) *Coriobacteriaceae_UCG_002* (**G**) *Faecalibaculum* (**H**) *Dubosiella* and (**I**) *Escherichia_Shigella*. Results are presented as means ± standard error of the mean (SEM) (n = 6). a *p* < 0.05, compared to the SHAM group. b *p* < 0.05, compared to the OVX group; c *p* < 0.05, compared to the E2 group.

The top ten phyla and top fifteen genera of bacterial communities from different groups are presented in Fig. 7A,B. Figure 7C–I showed the genus-levels abundance of the bacterial communities. The OVX group displayed a significantly lower abundance of *Lachnospiraceae_NK4A136_group*, *Faecalibaculum, Dubosiella* and *Coriobacteriaceae_UCG_002* compared to the E2 group (*p* < 0.05). Relative abundance of *Lachnospiraceae_NK4A136_group* in the LYPEP group was significantly higher and *Escherichia_Shigella* in YPEP groups was markedly lower, compared to the OVX group (*p* < 0.05).

### Correlation analysis

As displayed in Table 3, the abundance of *Faecalibaculum* and *Coriobacteriaceae_UCG_002* displayed negative correlations with serum TRAP, serum CTX-I, TV and positive correlations with serum Ca and BGP (*p* < 0.05). In addition, the abundance of *Coriobacteriaceae_UCG_002* displayed positive correlations with serum BMD and Tb.N (*p* < 0.05). The abundance of *Dubosiella* displayed a negative correlation with serum CTX-I, while the abundance of *unclassified_Lachnospiraceae* showed a positive correlation with Tb.Th (*p* < 0.05). There was no significant difference between groups of other intestinal flora of the genera.

**Table 3:** Spearman’s correlation between gut microbiota at the genus level with bone serum and microstructure parameters.

	BALP	ALP	TRAP	CTX-I	Ca	P	BGP	BMD	TV	BV	BV/TV	Tb.Th	Tb.Sp	Tb.N
*Alloprevotella*	0.19	− 0.19	0.23	0.12	0.01	0.25	− 0.10	− 0.34	0.06	− 0.18	− 0.05	− 0.36	0.14	− 0.29
*unclassified_Oscillospiraceae*	− 0.21	0.03	− 0.02	0.19	− 0.17	− 0.10	− 0.16	− 0.15	0.05	0.12	0.26	− 0.15	− 0.13	−.071
*Lachnospiraceae_NK4A136_group*	0.01	− 0.06	− 0.16	− 0.11	0.08	− 0.08	0.09	0.17	− 0.15	0.17	0.35	0.25	− 0.08	0.22
*unclassified_Lachnospiraceae*	0.06	0.10	− 0.08	− 0.06	− 0.08	0.17	− 0.11	− 0.16	0.40	0.20	− 0.26	0.59*	0.07	− 0.31
*Faecalibaculum*	− 0.31	− 0.20	− 0.38*	− 0.65**	0.40*	− 0.03	0.40*	0.15	− 0.45*	− 0.22	0.11	0.13	− 0.13	0.27
*Coriobacteriaceae_UCG_002*	− 0.13	− 0.34	− 0.50**	− 0.71**	0.51**	0.08	0.46*	0.53*	− 0.49*	− 0.01	0.30	0.22	− 0.33	0.63*
*Dubosiella*	− 0.28	0.00	− 0.28	− 0.38*	0.29	− 0.13	0.27	0.16	− 0.44	− 0.25	0.01	0.13	− 0.18	0.26
*Lactobacillus*	0.03	0.13	0.01	− 0.01	− 0.09	0.31	0.21	− 0.35	− 0.04	− 0.40	− 0.24	− 0.25	0.37	− 0.25

BALP, Bone alkaline phosphatase; ALP, alkaline phosphatase; TRAP, tartrate-resistant acid phosphatase; CTX-I, C-terminal telopeptide of type I collagen; Ca, calcium; P, phosphorus; BGP, bone gla protein; BMD, bone mineral density; TV, tissue volume; BV, bone volume; BV/TV, bone volume fraction; Tb.Th, trabecular thickness; Tb.Sp, trabecular separation; Tb.N, trabecular number. *p < 0.05; **p < 0.01.

## Discussion

YPEP is a bioactive peptide extracted from egg yolk and has been reported to promote bone formation^10,11^. Consistent with previous studies, we found that the bone formation rate of OVX group was significantly lower than bone resorption, and bone turnover accelerated, resulting in a large amount of bone loss^26^. Serum ALP, BALP, BGP, TRAP and CTX-I are considered specific and sensitive markers of bone turnover, which can reveal the pathogenesis of metabolic bone disease^27–29^. Serum BALP and BGP are considered as specific and sensitive markers for bone formation^27^. The bioactive peptides, such as yak bone collagen peptides and phosphorylated peptides from Antarctic krill Euphausia superba had been reported to alter bone turnover markers in OVX rats^8,30^. As for YPEP, a previous study analyzed its effects on some bone metabolic markers (ALP, CTX), but no significant differences were found between the OVX group and the YPEP group^11^. In addition, the effects of YPEP on serum BALP and TRAP in OVX rats have not been reported. In the present study, compared with the OVX group, YPEP groups had significantly higher serum levels of BGP and BALP. Simultaneously YPEP treatment also reduced the levels of TRAP and CTX-I in serum, which are generally considered as markers of osteoclasts during bone resorption^29,30^. BMD is the gold standard for evaluating the risk of osteoporosis^31^. The abovementioned study showed that BMD in YPEP groups was markedly higher than in OVX group, but bone microstructure parameters were not analyzed^11^. In the present study, in addition to BMD, we also analyzed the parameters of the femoral microstructure. Compared to the the OVX group, the LYPEP and HYPEP groups had markedly lower Tb.Sp, as well as higher BMD, BV/TV and Tb.N of the femurs. These results indicated that YPEP had potential protective effects on preventing bone loss in OVX-induced osteoporosis rats.

As confirmed by previous studies, Wnt/β-catenin signaling pathway plays a key role in skeletal development and homeostasis and is a potential target for osteoporosis treatment^32,33^. Duck egg white–derived peptide had been shown to play an anti-osteoporosis role in OVX-induced rats by activating the Wnt/β-catenin signaling pathway^16^. An in vitro experiment also demonstrated that the desalted duck egg white peptides could activate the Wnt/β-catenin signaling pathway, promoting the expression of related proteins, such as Wnt3a, LRP5, β-catenin and RUNX2^9^. However, no studies have confirmed that YPEP can improve osteoporosis by activating the Wnt/β-catenin signaling pathway. In the present study, we found for the first time that the protein levels of Wnt3a, β-catenin and LRP5 were significantly higher in YPEP groups than in the OVX group. Activation of Wnt/β-catenin signaling may promote osteogenesis and osteoblast differentiation by inducing RUNX2 expression^34^. RUNX2 is an important regulator in the differentiation of mesenchymal stem cells into osteoblasts and can promote the maturation of osteoblasts^35^. A previous in vivo experiment found that the novel soy peptide induced osteoblast differentiation via activating RUNX2^36^. Our study found that the protein level of RUNX2 was higher in the YPEP groups than in the OVX group, suggesting an enhancement in osteoblast differentiation and osteogenic activity. OPG is produced by mature osteoblasts, which mirrors the activity and osteogenic ability of osteoblasts^37^. In previous studies, the relationship between OPG and osteoporosis was contradictory. A cohort study found that serum levels of OPG were positively correlated with BMD^38^. However, a cross-sectional study found a significant negative association between serum OPG and femoral BMD^39^. In our study, compared with the SHAM group, the OVX group had a significantly lower protein level of OPG. The protein levels of OPG in the YPEP groups were significantly higher than in the OVX group. Bone is a complex tissue populated by a highly heterogeneous mix of cell types (osteocytes, osteoblasts, osteoclasts, cartilage, bone marrow cells, etc.) in different compartments^40,41^. In the study, the femoral metaphysis was collected for western blotting, the protein expression profile presented could be a mixture of osteoblasts and bone marrow cells^42^. This may be a limitation of the study.

Contrary to initial hypothesis, the improving effect of YPEP on osteoporosis seems to have no relationship with intestinal flora. Gut microbiota plays an important role in bone homeostasis^43^. Previous study found that OVX surgery led to significant changes in the composition of intestinal microbes at 14 weeks later, such as increased F/B ratio, Clostridia and Melainabacteria, and decreased Bacteroidia^44^. Altered gut microbiota in OVX model was also observed at 12 weeks later after surgery in another study^45^. However, in the present study, no significant difference was observed in the composition of gut microbiota between OVX and SHAM groups. Possible reasons for this inconsistency are as follows. On one hand, gut microbiota composition was determined at 8 weeks later after OVX surgery. The shorter duration in the present study may lead to a much smaller changes of intestinal flora than previous studies^44,45^. On the other hand, difference in animals (such as age) and environment (such as season) may also influence intestinal flora^46,47^. In the present study, although the relative abundance of *Lachnospiraceae_NK4A136_group* and *Escherichia_Shigella* was significantly different between OVX group and YPEP groups, no significant correlation was found between these bacteria and parameters related to bone metabolism. To our knowledge, no other studies have evaluated the effect of YPEP on gut microbiota in osteoporosis models, and studies are needed to verify this result.

## Conclusion

**Figure 8:**
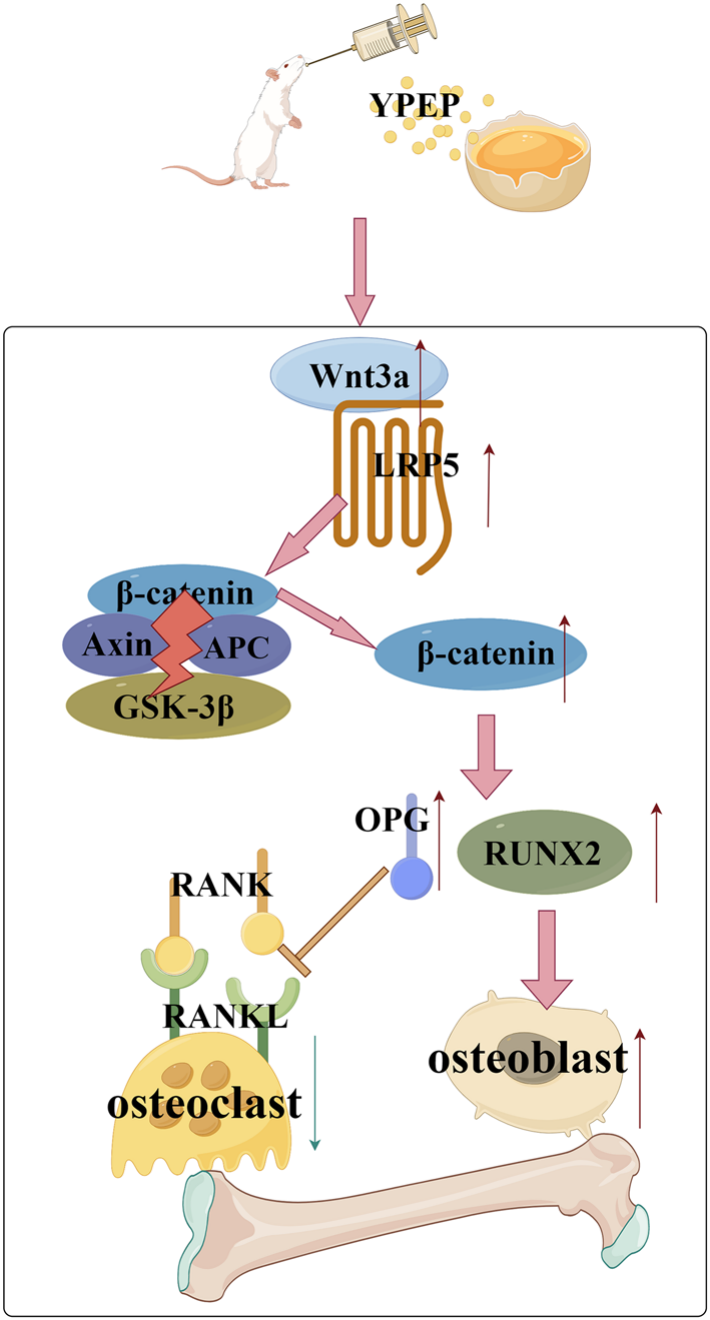
Potential mechanisms underlying the preventive effect of YPEP on OVX-induced osteoporosis.

In conclusion, YPEP supplementation can prevent bone loss in OVX-induced osteoporosis rats, and the mechanism may be associated with its modulating effect on Wnt/β-catenin signaling pathway (Fig. 8).

### Supplementary Information

Supplementary Figure 1.

Supplementary Figure 2.

## Data Availability

The datasets generated during and/or analyzed during the current study are available from the corresponding author upon reasonable request.
