# Supplementary figures and images for: Hydrolyzed egg yolk peptide prevented osteoporosis by regulating Wnt/β-catenin signaling pathway in ovariectomized rats
Source: Sci Rep. 2024 May 3;14:10227. doi: 10.1038/s41598-024-60514-8 (PMC11068896; doi:10.1038/s41598-024-60514-8)

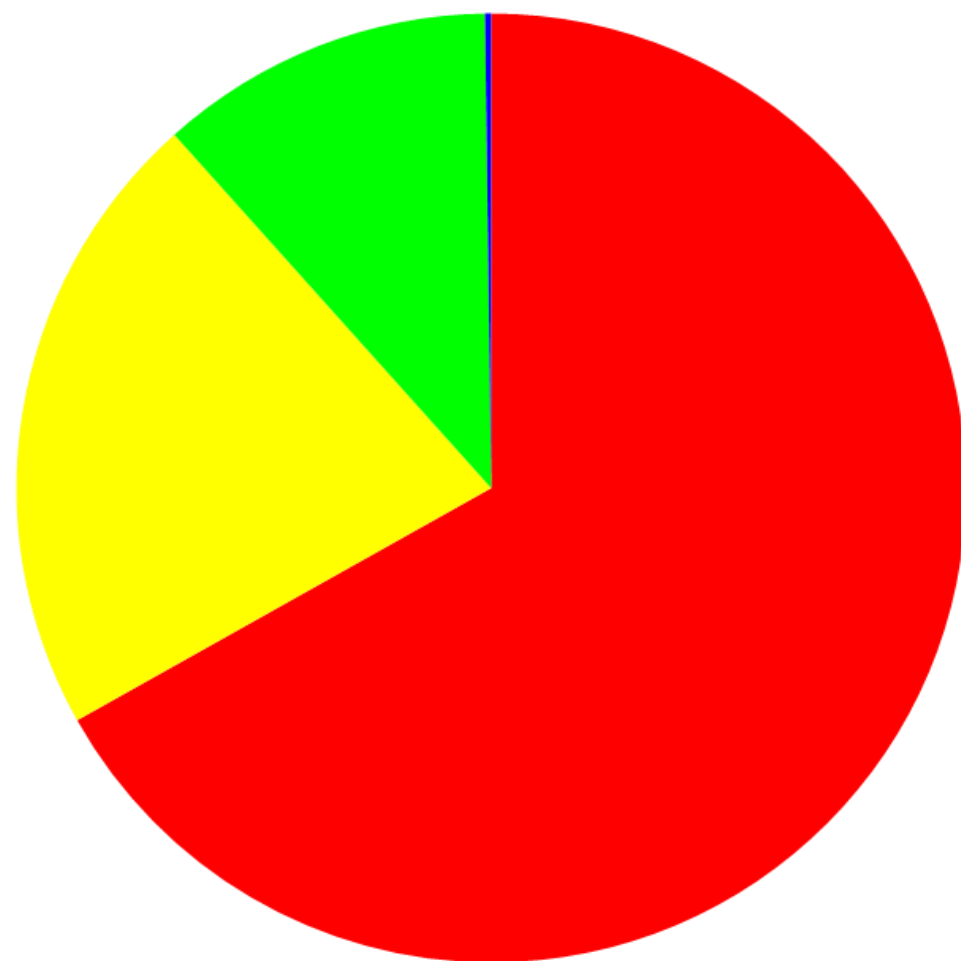

- 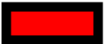 100-500 Da 66.86%
- 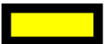 500-1000 Da 21.51%
- 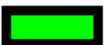 1000-5000 Da 11.42%
- 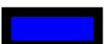 ≥5000 Da 0.21%

**total =100%**

Supplement: Supplementary file 1 — Supplementary Figure 1. [file 41598_2024_60514_MOESM1_ESM.pdf]
