# Supplementary material for: Hydrolyzed egg yolk peptide prevented osteoporosis by regulating Wnt/β-catenin signaling pathway in ovariectomized rats
Source: Sci Rep. 2024 May 3;14:10227. doi: 10.1038/s41598-024-60514-8 (PMC11068896; doi:10.1038/s41598-024-60514-8)
Supplement: Supplementary file 2 — Supplementary Figure 2. [file 41598_2024_60514_MOESM2_ESM.pdf]

β-actin -42KD

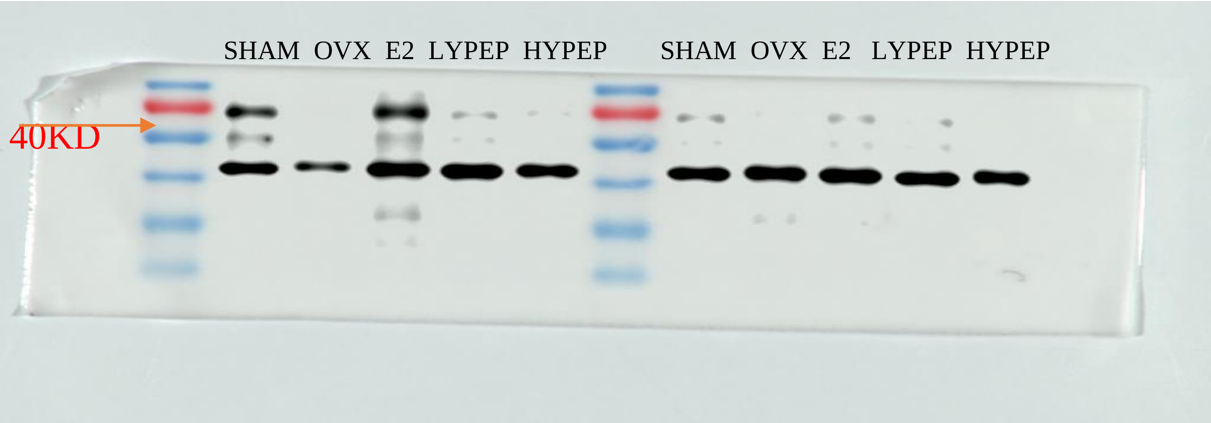

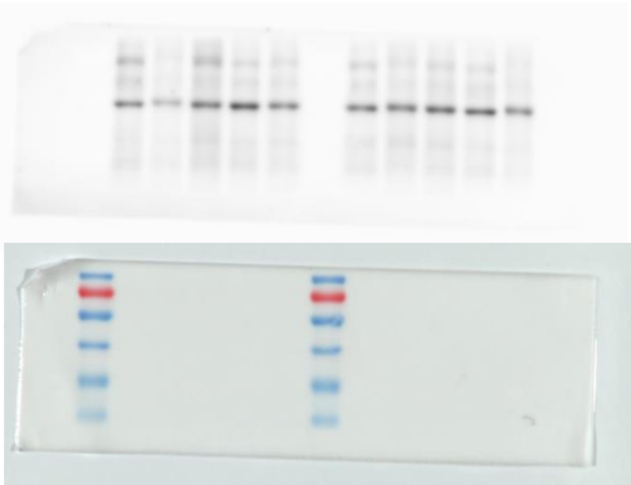

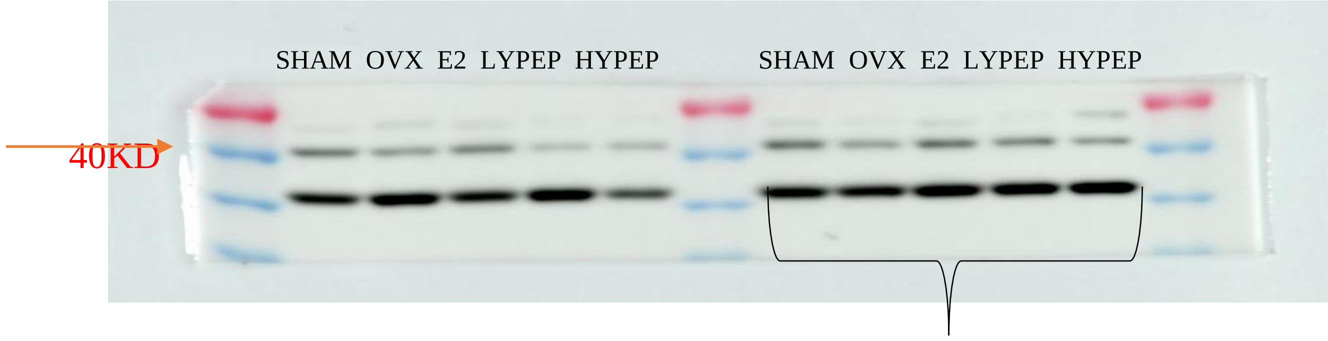

Used in other animal experiments

RUNX2-57KD

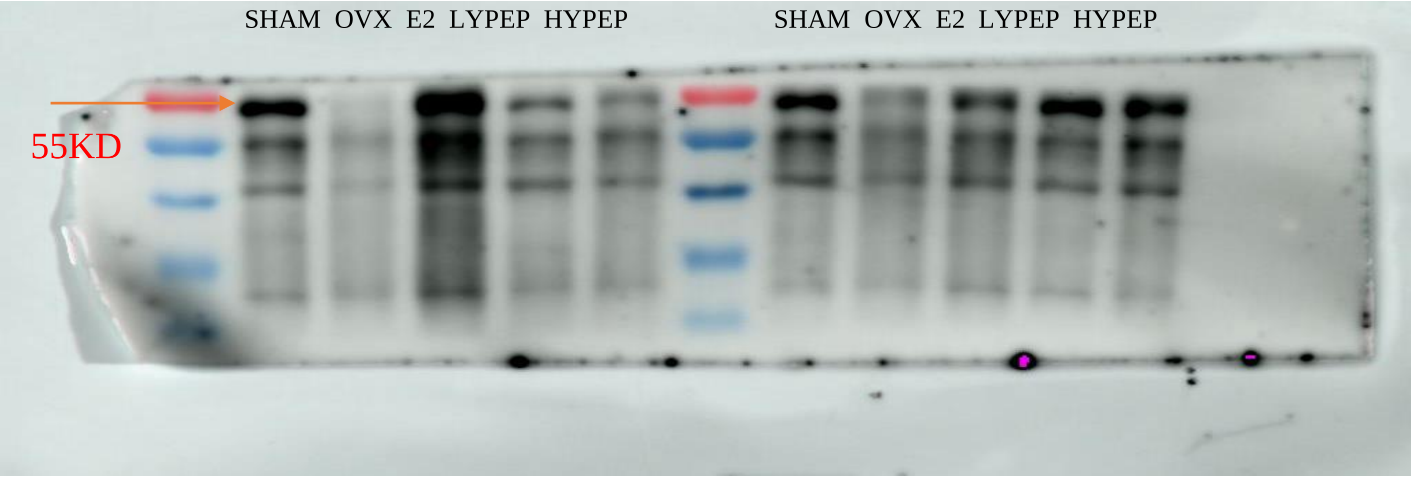

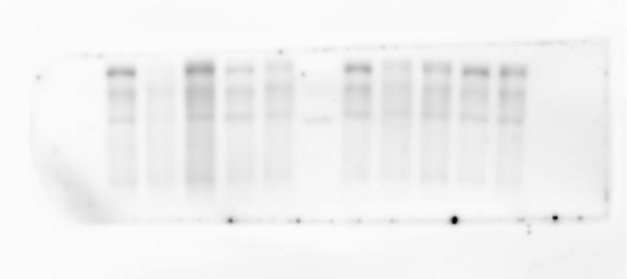

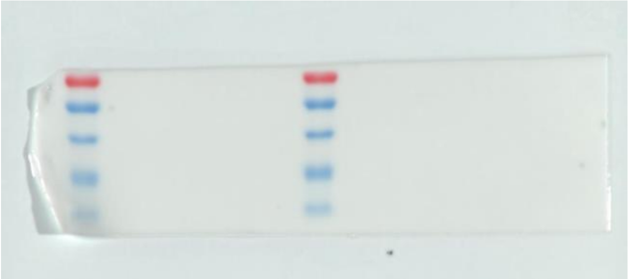

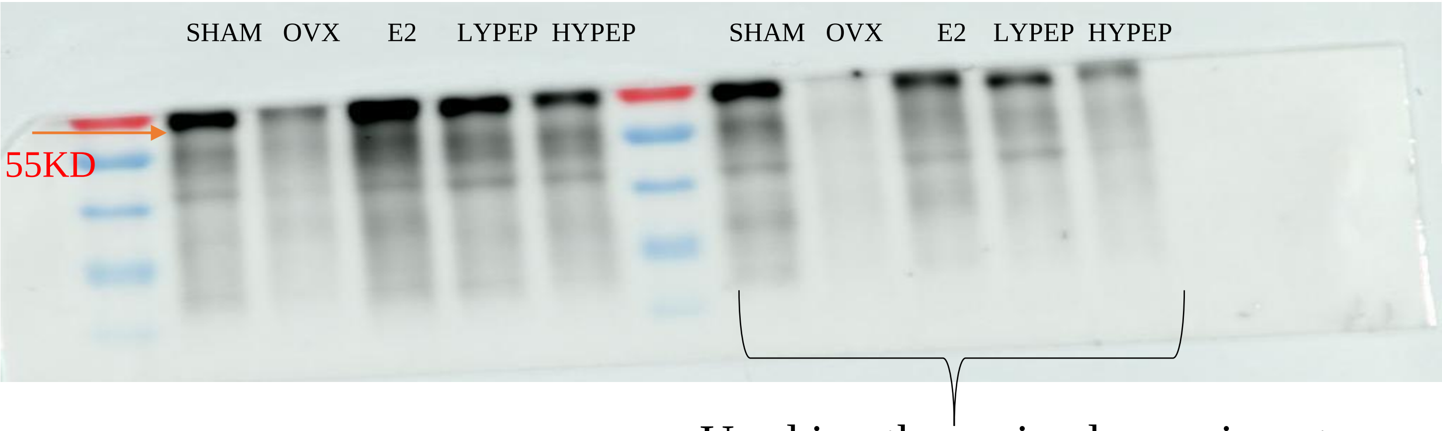

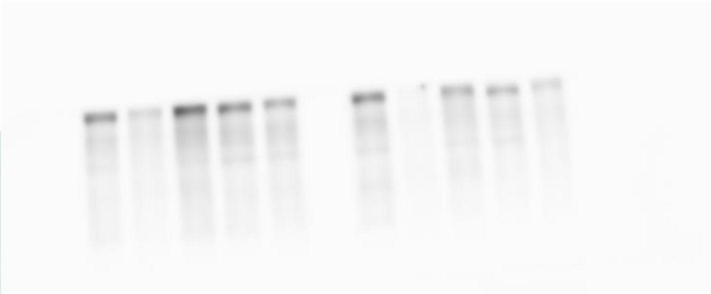

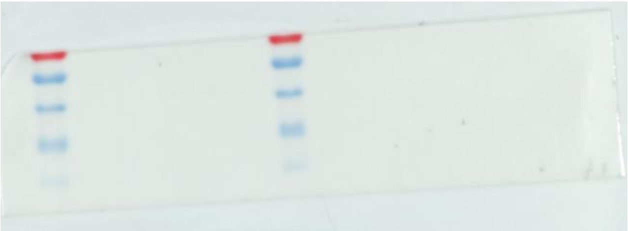

Used in other animal experiments

OPG-44KD

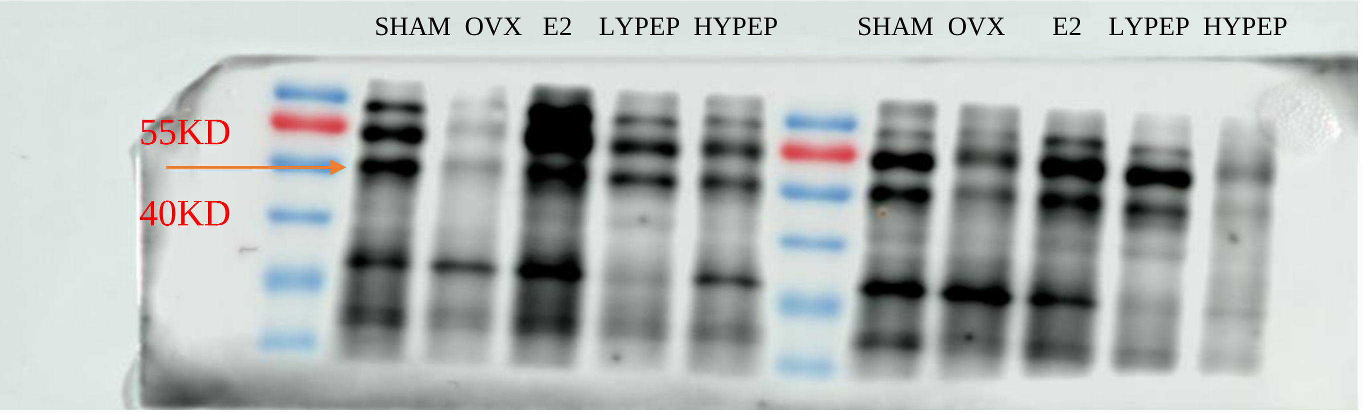
SHAM OVX E2 LYPEP HYPEP SHAM OVX E2 LYPEP HYPEP
55KD
40KD

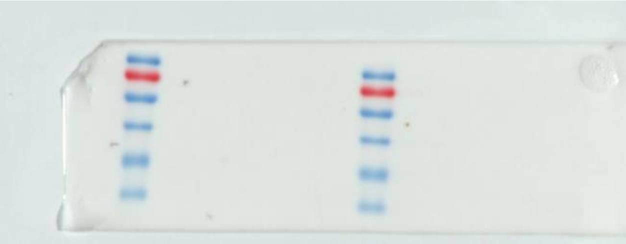

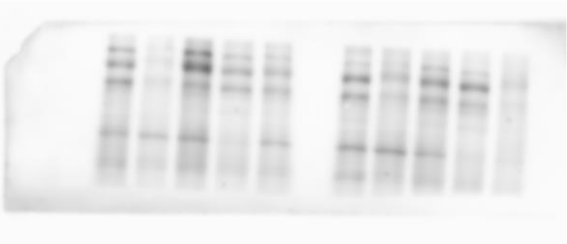

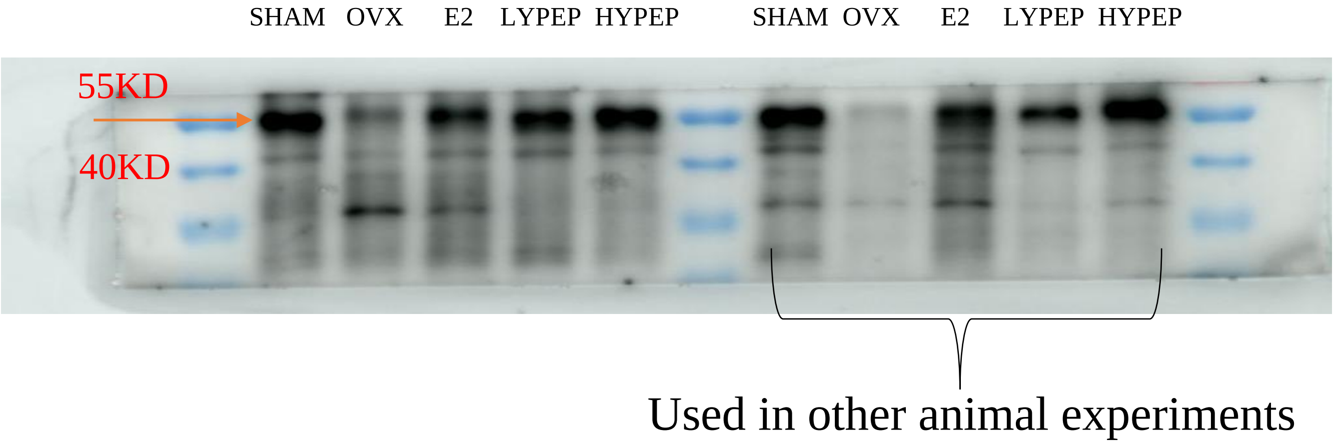
SHAM OVX E2 LYPEP HYPEP SHAM OVX E2 LYPEP HYPEP
55KD
40KD
Used in other animal experiments

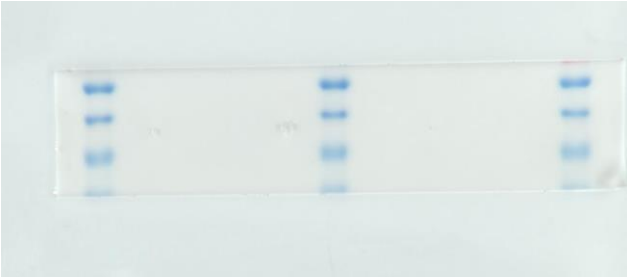

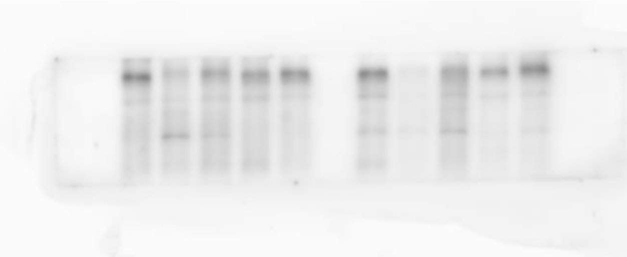

Wnt3a-39KD

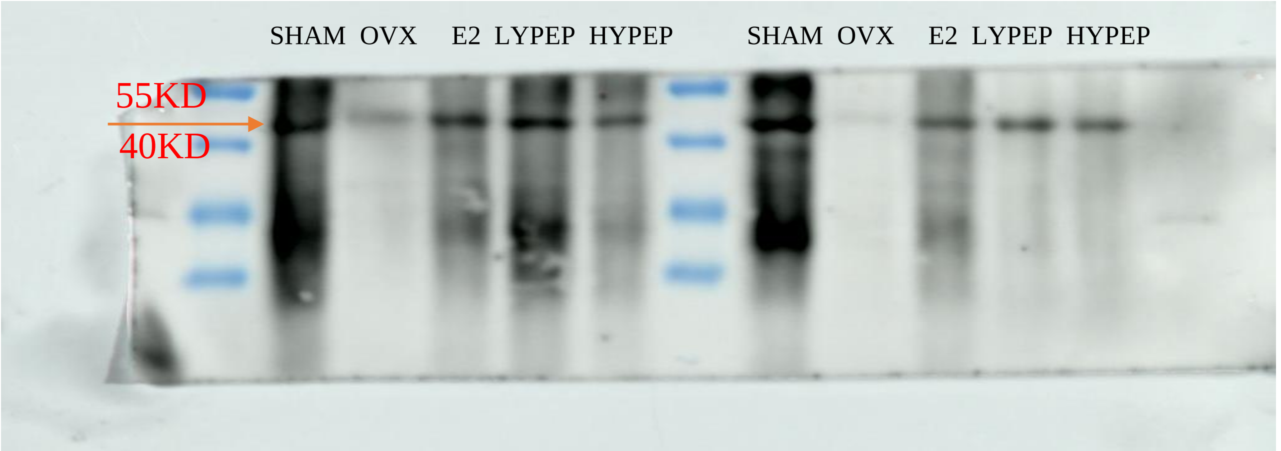

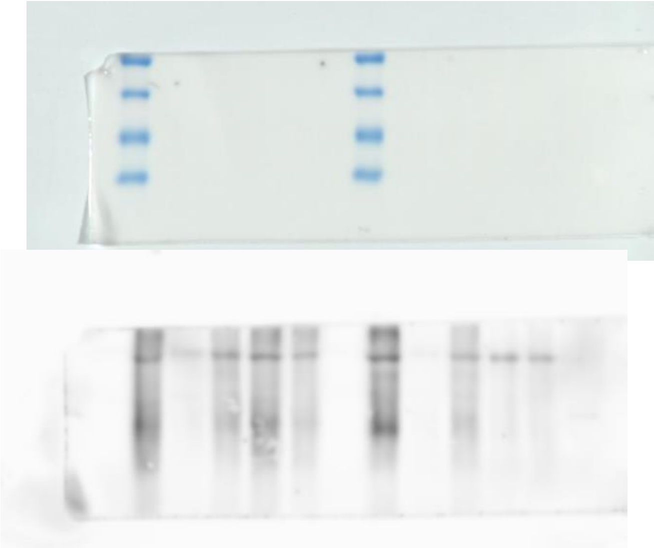

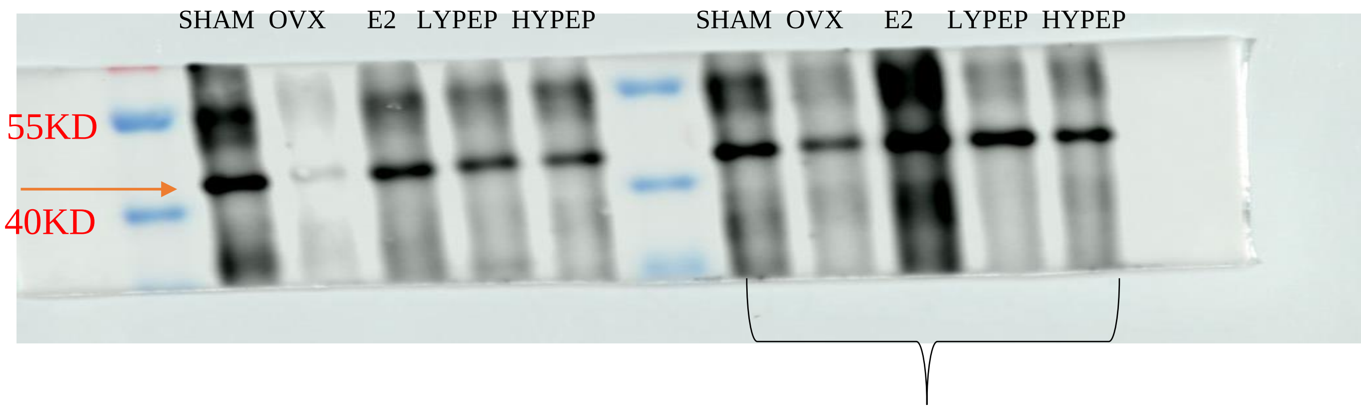

Used in other animal experiments

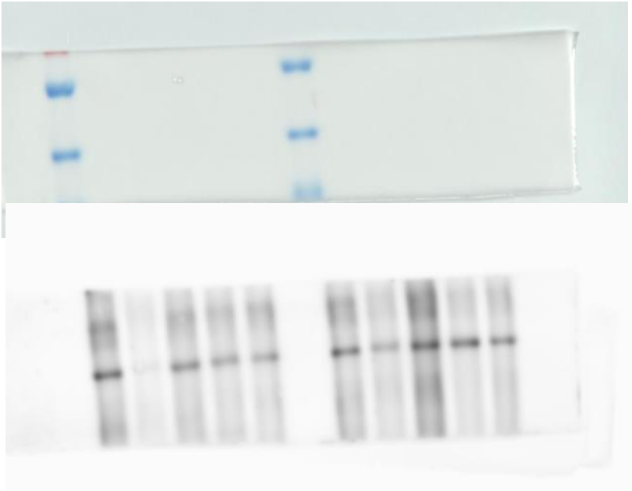

β-catenin-92KD

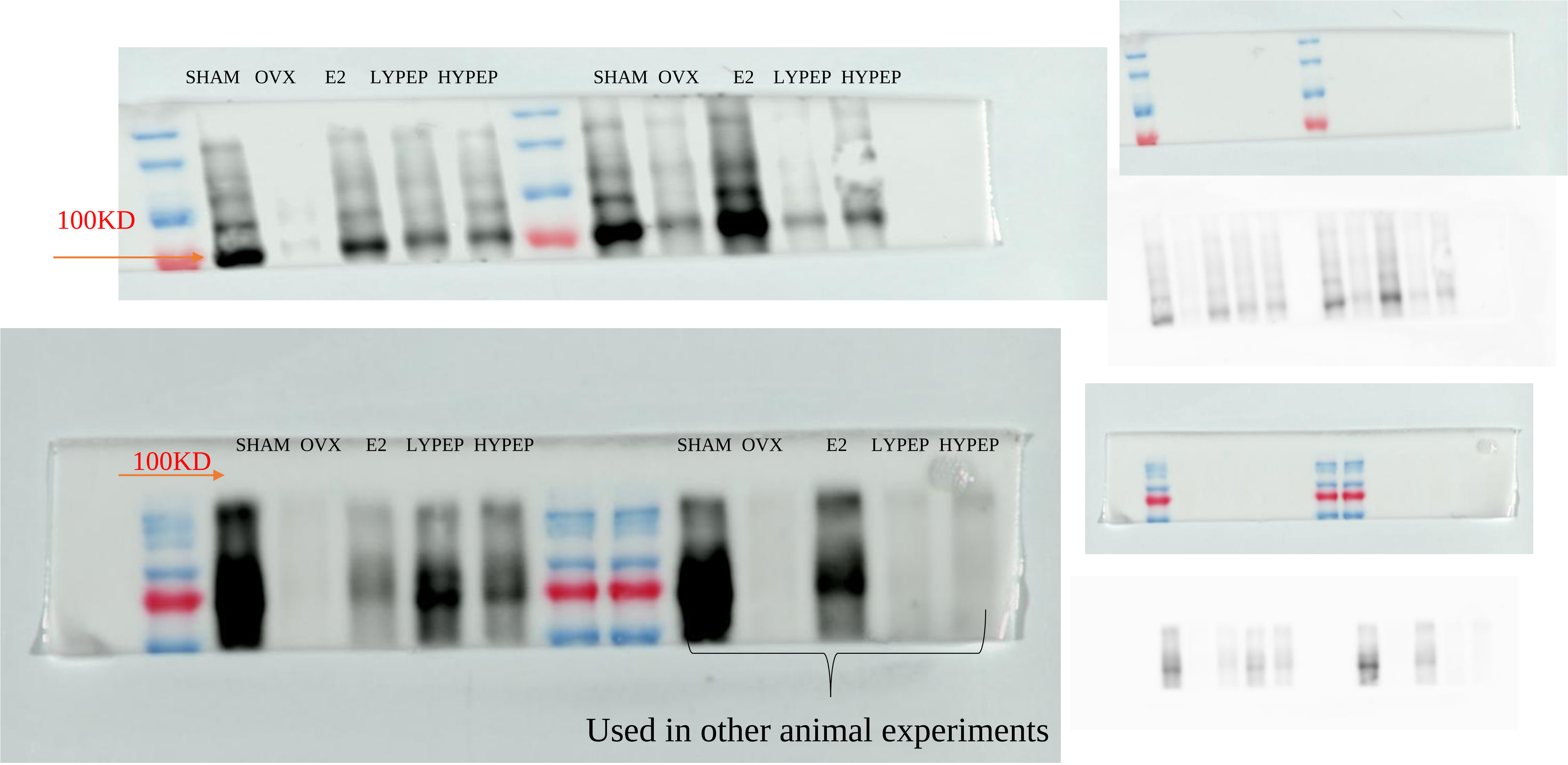

LRP5-179KD

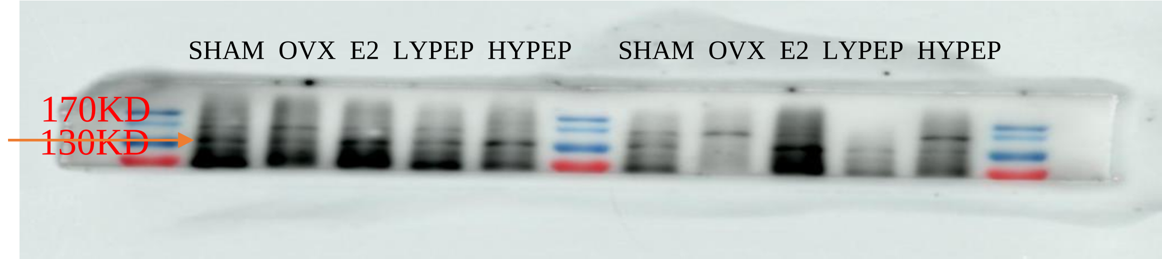

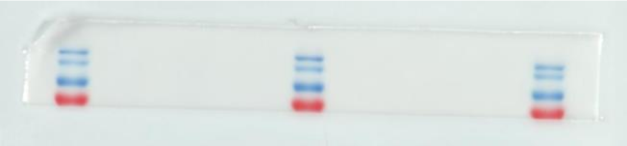

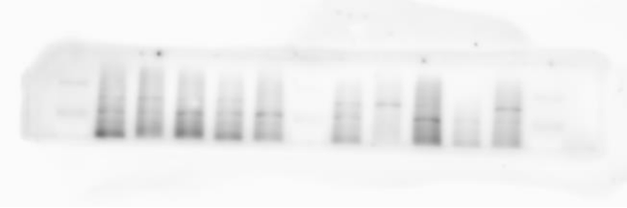

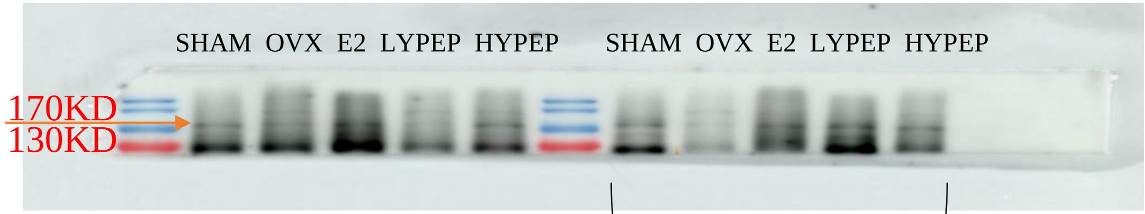

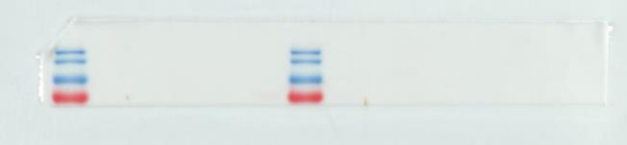

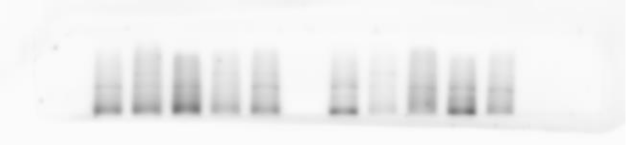

Used in other animal experiments

RANKL-39KD

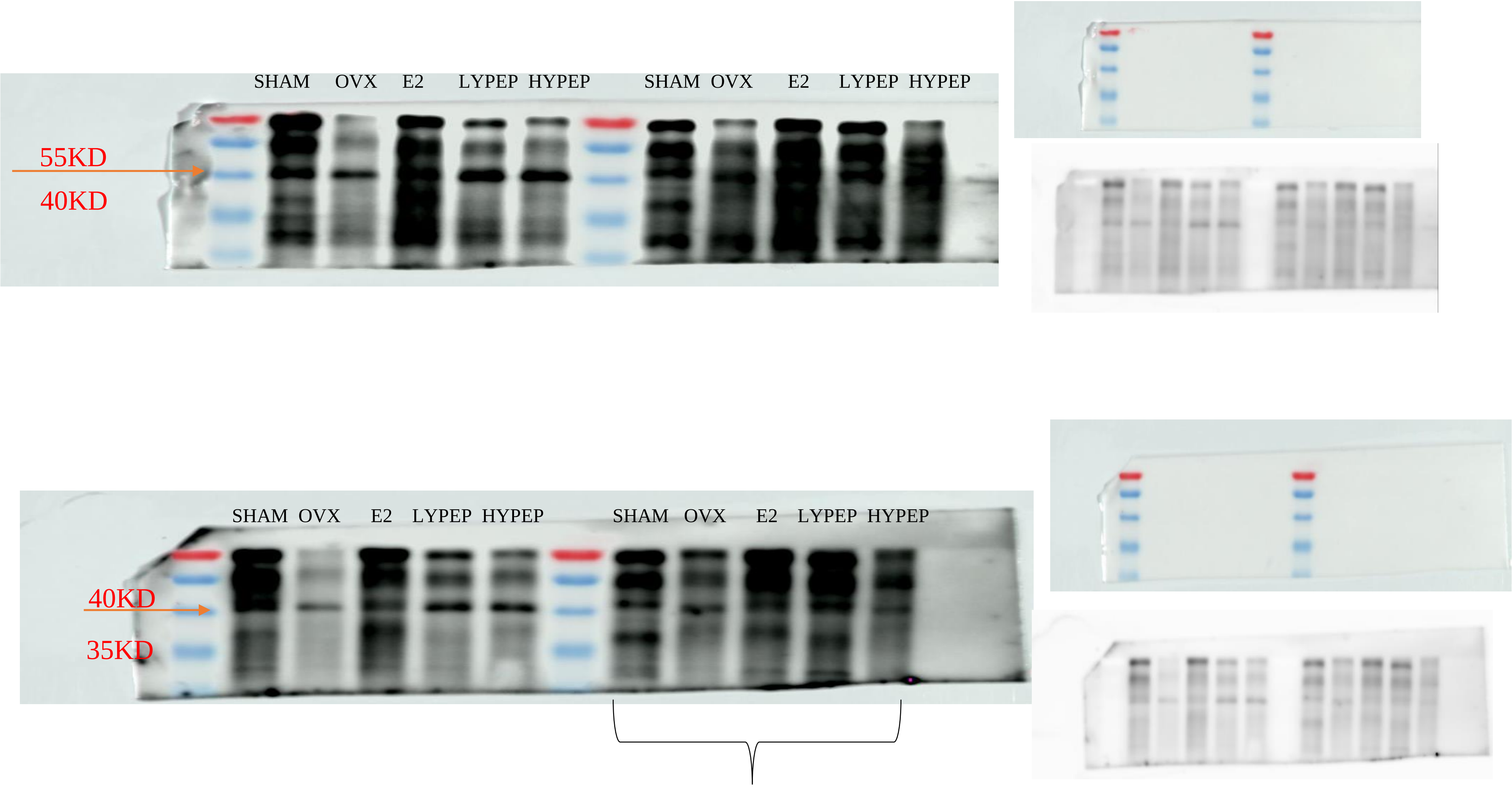

Used in other animal experiments
